# Patient-specific anatomical alignment relative to the contralateral collodiaphyseal angle as an independent predictor of screw cut-out after proximal femoral nailing

**DOI:** 10.1186/s13018-025-06537-x

**Published:** 2026-01-24

**Authors:** Ali Can Koluman, Basar Burak Cakmur, Altug Duramaz, Cemal Kural, Nezih Ziroglu

**Affiliations:** 1 Department of Orthopedics and Traumatology, Bakirkoy Dr. Sadi Konuk Training and Research Hospital, 34147 Istanbul, Turkey; 2 Department of Orthopedics and Traumatology, Beylikduzu State Hospital, 34500 Istanbul, Turkey; 3 Department of Orthopedic and Traumatology, Acibadem University Atakent Hospital, 34303 Istanbul, Turkey; 4 Department of Orthopedic Prosthetics and Orthotics, Acibadem Mehmet Ali Aydınlar University, Vocational School of Health Services, 34303 Istanbul, Turkey

**Keywords:** Barthel index, Collodiaphyseal angle, Functional outcome, Intertrochanteric fracture, Patient-specific alignment, Proximal femoral nailing, Screw cut-out, Tip–apex distance

## Abstract

**Background:**

Screw cut-out remains a major mechanical complication after proximal femoral nailing (PFN) for intertrochanteric fractures. Traditional predictors such as tip–apex distance (TAD) and reduction quality do not account for individual femoral anatomy. This study aimed to determine whether deviation from the contralateral collodiaphyseal (CCD) angle (|Δ angle|) independently predicts screw cut-out after PFN.

**Methods:**

A total of 354 patients (mean age 77.6 ± 12.0 years; 58% female) treated with PFN between 2015 and 2020 were retrospectively analyzed. Radiographic parameters included TAD and the absolute difference between postoperative and contralateral collodiaphyseal (CCD) angles (|Δ angle|), representing patient-specific alignment. Functional outcomes were assessed using the Harris Hip Score, Barthel Index, and time to full weight bearing. Univariate and multivariable logistic regression analyses were performed to identify independent predictors of screw cut-out.

**Results:**

Screw cut-out occurred in 56 patients (15.8%) and was associated with larger TAD, greater |Δ angle| deviation, and poorer reduction quality (all *p* < 0.01). In multivariable analysis, TAD, |Δ angle|, and reduction quality independently predicted cut-out. Patients with cut-out exhibited lower functional scores and delayed weight bearing, indicating substantial impairment in postoperative recovery.

**Conclusion:**

Patient-specific anatomical alignment, along with TAD and reduction quality, independently predicts screw cut-out. Deviations ≥ 9° from native CCD alignment increase mechanical failure risk and delay functional recovery.

## Introduction

Intertrochanteric femoral fractures are among the most common injuries in the elderly and are associated with loss of independence, reduced quality of life, and increased mortality. Proximal femoral nailing (PFN) has become the standard treatment due to its biomechanical stability and potential for early mobilization. However, mechanical complications—particularly screw cut-out through the femoral head—remain a major concern, often leading to revision arthroplasty and prolonged rehabilitation [1].

Hip fractures represent a major public health burden worldwide, with incidence increasing due to population aging and osteoporosis-related fragility. Despite significant improvements in surgical techniques and implant design, recent registry-based and multicenter studies have reported reoperation rates between 8 and 15% after proximal femoral nailing, primarily due to mechanical complications such as screw migration and cut-out [2–6].

Numerous studies have investigated radiographic risk factors for cut-out. Among these, the tip–apex distance (TAD) and reduction quality are consistently identified as the strongest predictors of fixation failure [7–11]. Other parameters such as fracture morphology, implant design, bone mineral density, and particularly reduction quality have also been implicated in mechanical failure [9–11]. Several scoring systems combining these parameters have been proposed, including that of Kulakoğlu et al., which integrates TAD, CalTAD, reduction quality, and patient sex [12, 13]. However, these approaches are based on generic radiographic metrics and overlook individual anatomical variation.

A major limitation in existing literature is the use of a fixed target neck–shaft angle—typically around 130°—when assessing postoperative alignment [14, 15]. Although varus malreduction is known to increase failure risk, this fixed-angle approach neglects natural variation in femoral anatomy. To achieve more accurate evaluation, several authors have suggested using the contralateral femur as an anatomical reference, as side-to-side differences in CCD angle are usually minimal (< 5° [8–11]). This method allows a more patient-specific assessment of reduction, but its quantitative predictive value for screw cut-out has not been adequately validated.

Recent studies published in 2024–2025 have further emphasized that even with modern cephalomedullary systems, mechanical failure remains a relevant problem, particularly when reduction accuracy and implant positioning deviate from the optimal biomechanical alignment [16–20]. These findings underscore the need for patient-specific assessment of reduction quality and alignment parameters to reduce fixation-related complications.

Furthermore, while radiographic predictors have been extensively studied, the functional consequences of screw cut-out remain underexplored. This complication leads to pain, loss of mobility, and delayed rehabilitation—particularly detrimental in elderly patients [21–23]. Functional metrics such as the Harris Hip Score and Barthel Index, which reflect daily living capacity, have rarely been incorporated into predictive models.

Therefore, the objective of this study was to evaluate whether patient-specific deviation from the contralateral CCD angle (|Δ angle|) predicts screw cut-out after PFN. A secondary aim was to examine its relationship with postoperative functional outcomes. We hypothesized that greater deviation from the native CCD alignment would be independently associated with a higher risk of screw cut-out.

## Materials and methods

### Study design and patient selection

This retrospective cohort study included patients treated with proximal femoral nailing (PFN) for intertrochanteric femoral fractures between January 2015 and December 2020. A total of 354 consecutive patients met the inclusion criteria. Eligible patients were aged ≥ 18 years, had a unilateral fracture, were treated with a single PFN system, and had a minimum follow-up of 12 months. Patients with pathological fractures, multiple trauma, or inadequate radiographic or clinical follow-up were excluded. Demographic and clinical data, including age, sex, body mass index (BMI), DEXA T-score, and AO/OTA fracture type, were recorded for all patients. All patients had been ambulatory prior to injury. The study was approved by the Institutional Review Board (Approval No. 2022–06.06). Given the retrospective design and de-identified data, the requirement for informed consent was waived.

### Surgical technique

All procedures were performed on a radiolucent traction table under fluoroscopic control in both anteroposterior (AP) and lateral planes. Patients were positioned supine with the unaffected leg abducted to allow unobstructed imaging of the proximal femur. Closed reduction was attempted in all cases by applying gentle longitudinal traction combined with internal rotation until satisfactory alignment was achieved, as verified by fluoroscopy. If acceptable reduction could not be obtained, limited open reduction was performed through a small lateral incision.

A standard cephalomedullary nail system was used in all patients, featuring a proximal lag screw and an anti-rotation screw, with distal static locking to enhance rotational stability. The entry point was established at the tip of the greater trochanter, and the nail was inserted along the medullary canal under fluoroscopic guidance. The tip–apex distance (TAD) was routinely measured intraoperatively to ensure optimal implant positioning within the femoral head.

Postoperatively, a standardized rehabilitation protocol was followed. Quadriceps and ankle-pumping exercises were initiated on the first postoperative day. Patients were allowed to weight-bear as tolerated from postoperative day 1 using a walking aid, and progressed to full weight bearing once radiographic evidence of callus formation and clinical stability was achieved—typically within 6 to 8 weeks. Thromboprophylaxis and analgesic regimens were applied uniformly according to institutional guidelines.

### Radiographic evaluation

**Fig. 1:**
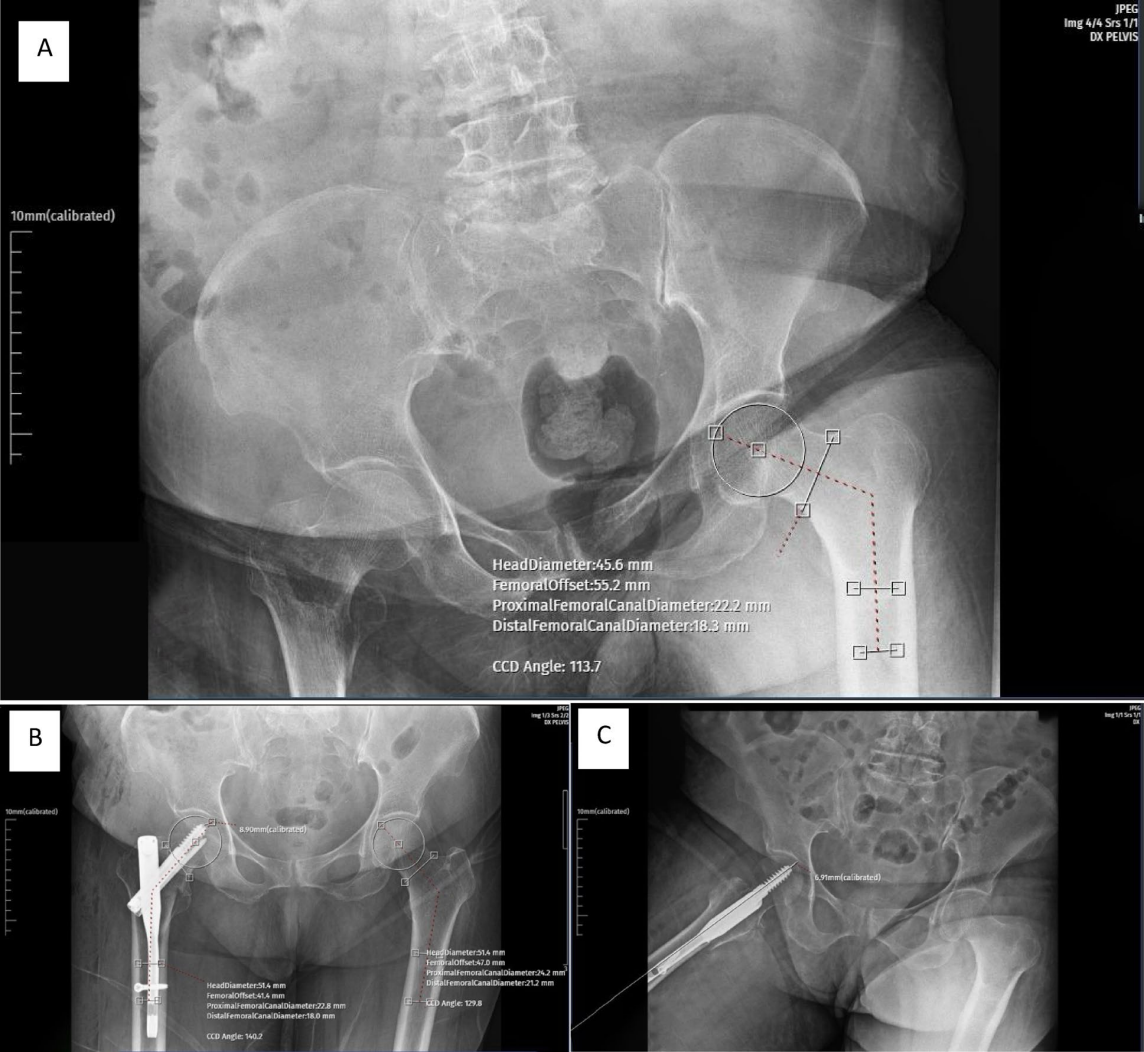
Representative radiographs from the same patient illustrating the measurement technique. **A** Preoperative anteroposterior pelvis view showing the intertrochanteric fracture and measurement of the contralateral (uninjured) collodiaphyseal (CCD) angle. Minor apparent varus deviation was observed in the contralateral measurement, likely due to suboptimal patient positioning related to fracture pain. **B** Immediate postoperative anteroposterior pelvis radiograph demonstrating improved neutral alignment, with postoperative and contralateral CCD measurements indicated. **C** Postoperative lateral pelvis radiograph illustrating determination of the tip–apex distance (TAD) according to Baumgaertner’s method. All measurements were performed on digitally calibrated images, and additional calibration was unnecessary because the PACS system automatically applied scaling

Fractures were classified preoperatively using the AO/OTA system (31-A1, 31-A2, 31-A3). Standardized anteroposterior (AP) and lateral hip radiographs were obtained postoperatively (Fig. 1). To minimize rotational bias, all radiographs were taken in a supine position with the patella facing upward; images showing excessive malrotation—defined by asymmetric visualization of the lesser trochanter—were excluded. Radiologic technologists followed a uniform acquisition protocol under senior supervision to ensure consistent image quality and magnification across all series.

- Tip–apex distance (TAD): calculated as described by Baumgaertner et al. [8].
- Collodiaphyseal (CCD) angle: defined as the angle between the femoral neck and diaphyseal axes. The postoperative CCD angle and the contralateral native CCD angle were measured using calibrated digital radiographs, and the absolute difference between them (|Δ angle|) was recorded as a patient-specific alignment parameter [21]. CCD angles were determined between the femoral neck axis and mid-diaphyseal axis using a digital goniometer tool within the PACS system. The contralateral reference value was obtained from the uninjured side on pre-injury or immediate postoperative pelvis radiographs, depending on image availability.
- Reduction quality was assessed using the *Baumgaertner Reduction Quality Criteria (BRQC)* [22]. Reduction was classified as good when both alignment and displacement criteria were met, acceptable when only one criterion was met, and poor when neither was met. Acceptable alignment was defined as a normal or slightly valgus neck–shaft angle on the AP view and < 20° angulation on the lateral view, with < 4 mm displacement on both planes. Anteromedial cortical continuity was verified on both AP and lateral radiographs to ensure mechanical stability, although it was not used as a separate grading criterion. This classical BRQC system remains widely used; recent updates have expanded its framework by emphasizing the role of anteromedial cortical support in reduction assessment [24]. The primary alignment variable was the absolute deviation between postoperative and contralateral CCD angles (|Δ angle|); the signed difference (Δ angle) was recorded only to indicate varus/valgus direction.

All radiographic measurements were performed by a single senior orthopedic surgeon experienced in hip trauma. Measurements were performed using standardized digital radiographs to minimize intraobserver variability. All linear measurements (including TAD) were performed on digitally calibrated radiographs in millimeters using the known lag screw diameter to account for magnification. Representative measurement examples are provided in Fig. 1, illustrating the method for determining postoperative and contralateral CCD angles, as well as the TAD measurement process.

### Functional evaluation

Functional outcomes were evaluated using the validated Turkish versions of the Harris Hip Score [25] and Barthel Index [26]. The time to full weight bearing (weeks) was recorded during routine follow-up visits. Clinical assessments were performed at 2 weeks, 6 weeks, 3 months, 6 months, 1 year, and annually thereafter. Functional scores were analyzed as secondary outcomes and were not included as predictors in the statistical models.

### Outcome definition

Patients were divided into two groups based on postoperative radiographs obtained during follow-up:

- Cut-out group: patients who developed radiographic evidence of lag screw cut-out through the femoral head.
- Control group: patients without evidence of cut-out or mechanical failure.

### Statistical analysis

All statistical analyses were conducted using NCSS 2007 (Kaysville, Utah, USA). Continuous variables were expressed as *mean* ± *standard deviation* or *median (interquartile range)*, and categorical variables as *frequency (percent)*. Normality was assessed using the Shapiro–Wilk test. Between-group comparisons were performed with the independent-samples t-test or Mann–Whitney U test for continuous variables and the chi-square or Fisher’s exact test for categorical variables. Variables with *p* < *0.10* in univariate analyses, as well as clinically relevant factors (TAD, |Δ angle|, reduction quality, BMI, DEXA T-score, age, and AO/OTA type), were entered into a multivariable logistic regression to determine independent associations with screw cut-out. Odds ratios (OR) with 95% confidence intervals (CI) were reported. All tests were two-sided, and *p* < *0.05* was considered statistically significant. To minimize model overfitting, the number of included predictors was limited to maintain an events-per-variable (EPV) ratio greater than 7, ensuring acceptable model stability. Post-hoc power analysis revealed an observed power of 0.82 for |Δ angle| and > 0.99 for TAD (α = 0.05), confirming adequate sensitivity of the regression model.

## Results

A total of 354 patients were included, comprising 298 without screw cut-out and 56 with cut-out during follow-up. The mean age of the entire cohort was 77.6 ± 12.0 years, and 58% were female. There were no significant between-group differences in age, sex distribution, BMI, or DEXA T-scores (all *p* > 0.05). Compared with controls, the cut-out group showed a greater absolute deviation from contralateral alignment (|Δ angle|, *p* < 0.01); the signed difference (Δ angle) was recorded for directionality only. The mean follow-up period was 24.3 ± 9.1 months (Table 1). Screw cut-out developed at a mean of 4.1 ± 1.8 months postoperatively (range, 1–6 months), and all cases occurred within the first 6 months after fixation.

**Table 1:** Univariate analysis of demographic, perioperative, radiological, and functional variables associated with screw cut-out

Variable	Control (n = 298)	Cut-out (n = 56)	*p* value
*Demographics*			
Age (years)	77.2 ± 12.8	79.6 ± 8.1	0.483
Sex (male/female)	131(44%) / 167(56%)	22(39%) / 34(61%)	0.559
BMI (kg/m^2^)	25.1 ± 3.5	25.4 ± 3.9	0.703
DEXA T-score (L1–L4)	− 2.09 ± 1.90	− 2.70 ± 0.79	0.255
*Injury characteristics*			
Side (right/left)	135 (45%) / 163(55%)	22 (39%)/ 34 (61%)	0.465
Mechanism of injury	Domestic: 152 (51%)	Domestic: 28 (50%)	0.603
	Simple fall: 129(43%)	Simple fall: 27(48%)	
	High fall: 5 (2%)	High fall: 0 (0%)	
	Traffic: 12 (4%)	Traffic: 1 (2%)	
Fracture type (AO/OTA)	A1: 131 (44%)	A1: 15 (27%)	**0.020**
	A2: 117 (39%)	A2: 33 (59%)	
	A3: 50 (17%)	A3: 8 (14%)	
*Perioperative variables*			
Type of anesthesia	General: 58 (19%)	20 (36%)	**0.013**
	Spinal: 240 (81%)	36 (64%)	
ASA class (I / II / III)	18 (6%) / 60 (20%)/ 220 (74%)	1 (2%) / 8 (14%) / 47 (84%)	0.216
Reduction method (closed/open)	278 (93%) / 20 (7%)	51 (91%) / 5 (9%)	0.569
Reduction quality (good / acceptable / poor)	176 (59%) / 108 (36%) / 14 (5%)	19 (34%) / 18 (32%) / 19 (34%)	** < 0.001**
Operative time (min)	85.6 ± 26.8	98.3 ± 29.7	**0.001**
Estimated blood loss (mL)	614.6 ± 914.7	1084.8 ± 1032.5	**0.001**
Postoperative infection	9 (3.0%)	3 (5.4%)	0.413
Revision surgery required	6 (2.0%)	51 (91.1%)	** < 0.001**
*Radiological parameters*			
Tip–apex distance (mm)	25.2 ± 7.8	33.0 ± 12.0	** < 0.001**
Δ angle(°) mean ± SD (range) (postop − contralateral CCD)	1.3 ± 5.9 (− 16 to 15)	1.3 ± 8.3 (− 16 to 16)	0.908
*Functional outcomes*			
Harris Hip score	76.3 ± 11.9	64.6 ± 6.3	** < 0.001**
Barthel index	87.9 ± 10.3	78.4 ± 9.3	** < 0.001**
Time to full weight bearing (weeks)	14.2 ± 1.7	16.5 ± 5.7	0.013

Values are presented as mean ± standard deviation, median (minimum–maximum), or number (percentage), as appropriate. Group comparisons were performed using the independent-samples t test or Mann–Whitney U test for continuous variables and the chi-square or Fisher’s exact test for categorical variables. Statistically significant *p* values (< 0.05) are shown in bold. Δ angle represents the signed difference between postoperative and contralateral CCD angles (positive = valgus, negative = varus). In contrast, |Δ angle| denotes the absolute magnitude of deviation and was the prespecified alignment variable used in the primary analyses and multivariable models

### Radiographic findings

**Fig. 2:**
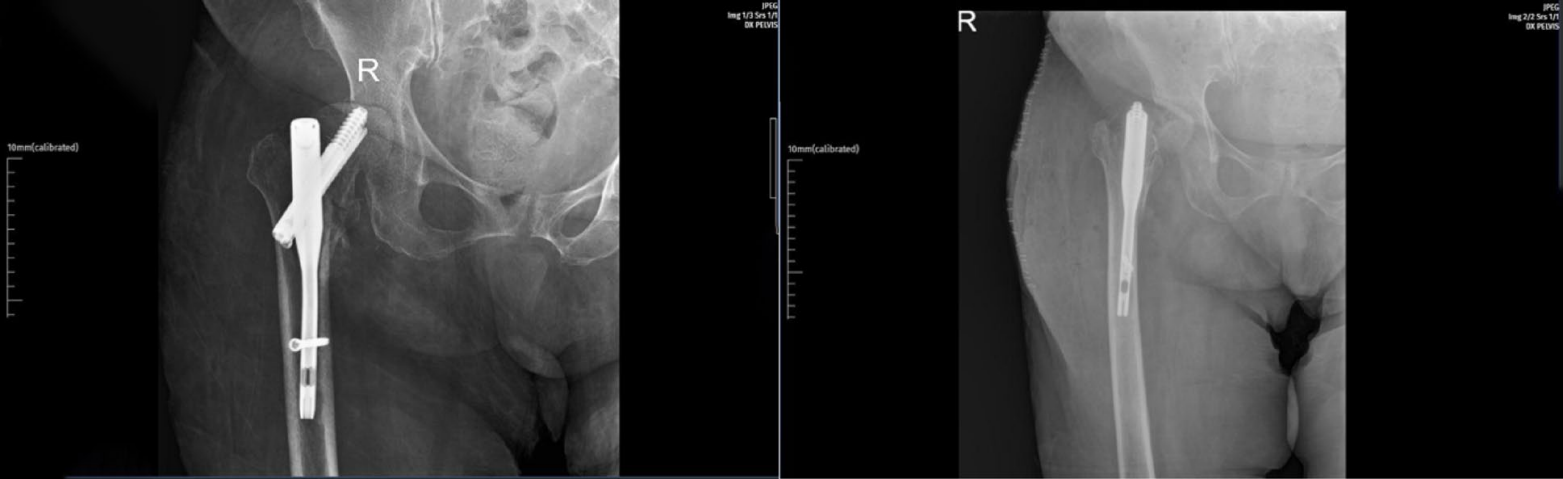
Postoperative anteroposterior pelvis radiographs from the same patient demonstrating the progression of mechanical failure. The left panel (3 months postoperatively) shows early screw cut-out through the femoral head with initial superior migration of the lag screw and partial loss of reduction. The right panel (6 months postoperatively) demonstrates further superior migration of the lag screw with complete loss of reduction and collapse of the fracture site. The depicted loss of femoral head sphericity represents mechanical collapse (screw cut-out) rather than avascular necrosis; no cases in this series demonstrated radiographic evidence of AVN

The mean tip–apex distance (TAD) was significantly greater in the cut-out group (33.0 ± 12.0 mm) compared with the control group (25.2 ± 7.8 mm, *p* < 0.001). The signed difference between postoperative and contralateral CCD angles (Δ angle) did not differ significantly between groups (*p* = 0.91). In contrast, the absolute deviation from the contralateral side (|Δ angle|) was significantly higher in patients who developed cut-out (*p* < 0.01). This discrepancy reflects that varus and valgus deviations in opposite directions cancel each other when analyzed as signed values, whereas |Δ angle| represents the true magnitude of malalignment irrespective of direction. A deviation of approximately 9° or more from the contralateral CCD angle was associated with an increased likelihood of cut-out (Fig. 2). Reduction quality was also significantly worse among patients with cut-out (*p* < 0.001). Both excessive varus and valgus alignment deviations were associated with mechanical instability. While valgus malalignment can alter load transfer and increase joint reaction forces, varus reduction exerts greater compressive and shear stress on the superior femoral head and is therefore considered biomechanically more detrimental. Although fracture type (AO/OTA classification) and anesthesia method showed between-group differences on univariate analysis, these factors did not remain significant in adjusted analyses.

Among patients who developed screw cut-out (n = 56), valgus malreduction (Δ angle >  + 5°) was observed in 16 cases (28.6%), whereas 13 cases (23.2%) exhibited varus deviation (Δ angle <  − 5°). The remaining 27 cases (48.2%) demonstrated near-neutral alignment (|Δ angle|≤ 5°). The maximum valgus deviation among cut-out cases was + 16°, and the maximum varus deviation was − 16°. Although both directional deviations were present, neither predominated overwhelmingly, suggesting that the magnitude rather than the direction of deviation was the principal determinant of mechanical failure.

### Multivariable analysis

**Fig. 3:**
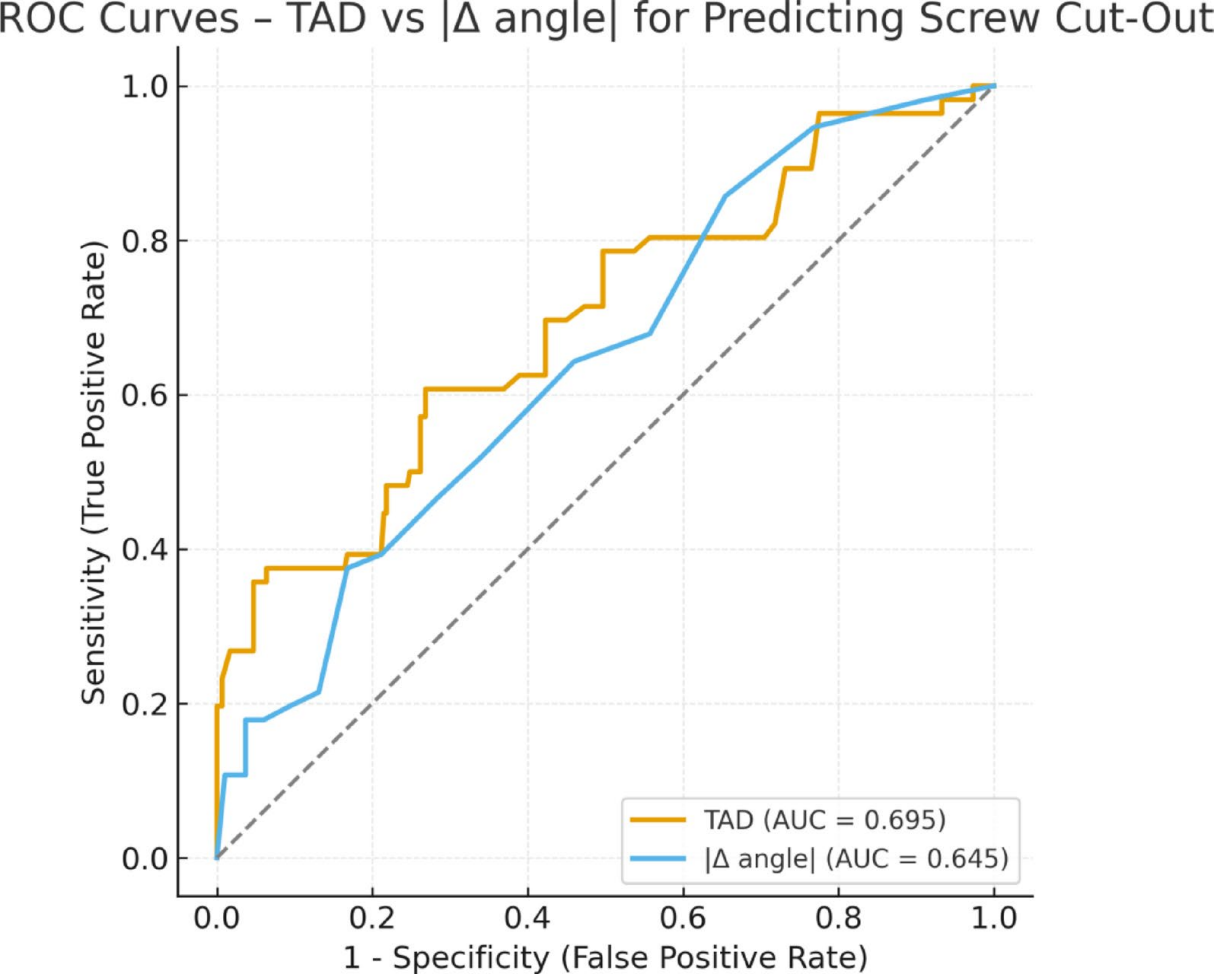
ROC curves of tip–apex distance (TAD) and absolute CCD deviation (|Δ angle|) for predicting screw cut-out. AUC was 0.695 for TAD and 0.645 for |Δ angle|, indicating moderate discriminative power for both parameters

In the multivariable logistic regression, TAD (per SD increase, OR 2.08; 95% CI 1.50–2.87; *p* < 0.001), |Δ angle| (per SD increase, OR 1.58; 95% CI 1.16–2.16; *p* = 0.004), and reduction quality (per one-step worse, OR 2.27; 95% CI 1.40–3.68; *p* = 0.001) were identified as independent factors associated with screw cut-out. BMI, DEXA T-score, age, fracture type, and anesthesia type were not independently related to mechanical failure (Table 2). The receiver operating characteristic (ROC) curves of TAD and |Δ angle| for predicting screw cut-out are presented in Fig. 3. Both parameters demonstrated acceptable discriminative ability (AUC = 0.695 for TAD and AUC = 0.645 for |Δ angle|), with TAD showing slightly higher predictive accuracy. Although the Hosmer–Lemeshow test reached borderline significance (*p* = 0.034), graphical calibration indicated acceptable agreement between predicted and observed probabilities (data not shown). Based on the ROC analysis, the optimal |Δ angle| cut-off value for predicting screw cut-out was 9.1°, yielding a sensitivity of 71.4% and specificity of 63.2% (Youden index = 0.346). This threshold was consistent with the visually observed deviation level associated with increased mechanical failure.

**Table 2:** Multivariate logistic regression analysis of factors associated with screw cut-out after proximal femoral nailing

Variable	Odds ratio (OR)	95% CI	*p* value
Tip–apex distance (per 1 SD increase)	2.08	1.50–2.87	** < 0.001**
Absolute CCD deviation, |Δ angle| (per 1 SD increase)	1.58	1.16–2.16	**0.004**
Reduction quality (per 1-step worse)	2.27	1.40–3.68	**0.001**
Body mass index (kg/m^2^)	1.20	0.88–1.64	0.258
DEXA T-score (L1–L4)	0.64	0.36–1.12	0.117
Age (years)	0.98	0.58–1.66	0.943
Fracture type (AO/OTA)	1.19	0.71–1.97	0.514

Continuous predictors were standardized (reported **per 1 SD increase**). **Δ angle** denotes the signed difference (postoperative − contralateral CCD), whereas **|Δ angle|** denotes the **absolute** deviation (patient-specific malalignment magnitude). Model calibration was acceptable (Hosmer–Lemeshow χ^2^ ≈ 16.68, df = 8, *p* ≈ 0.034). Discrimination: apparent AUC = 0.803; optimism-adjusted AUC (bootstrap, 200 resamples) = 0.778. Bold values indicate statistically significant differences

### Functional outcomes

Patients who experienced screw cut-out demonstrated significantly poorer functional outcomes. The mean Harris Hip Score was lower in the cut-out group (64.6 ± 6.3) than in controls (76.3 ± 11.9; *p* < 0.001; Cohen’s d = 1.17, large effect). Similarly, the Barthel Index was reduced in the cut-out group (78.4 ± 9.3 vs 87.9 ± 10.3, *p* < 0.001; Cohen’s d = 1.00, large effect), and the time to full weight bearing was significantly delayed (16.5 ± 5.7 vs 14.2 ± 1.7 weeks, *p* = 0.013).

### Revision procedures and complications

**Fig. 4:**
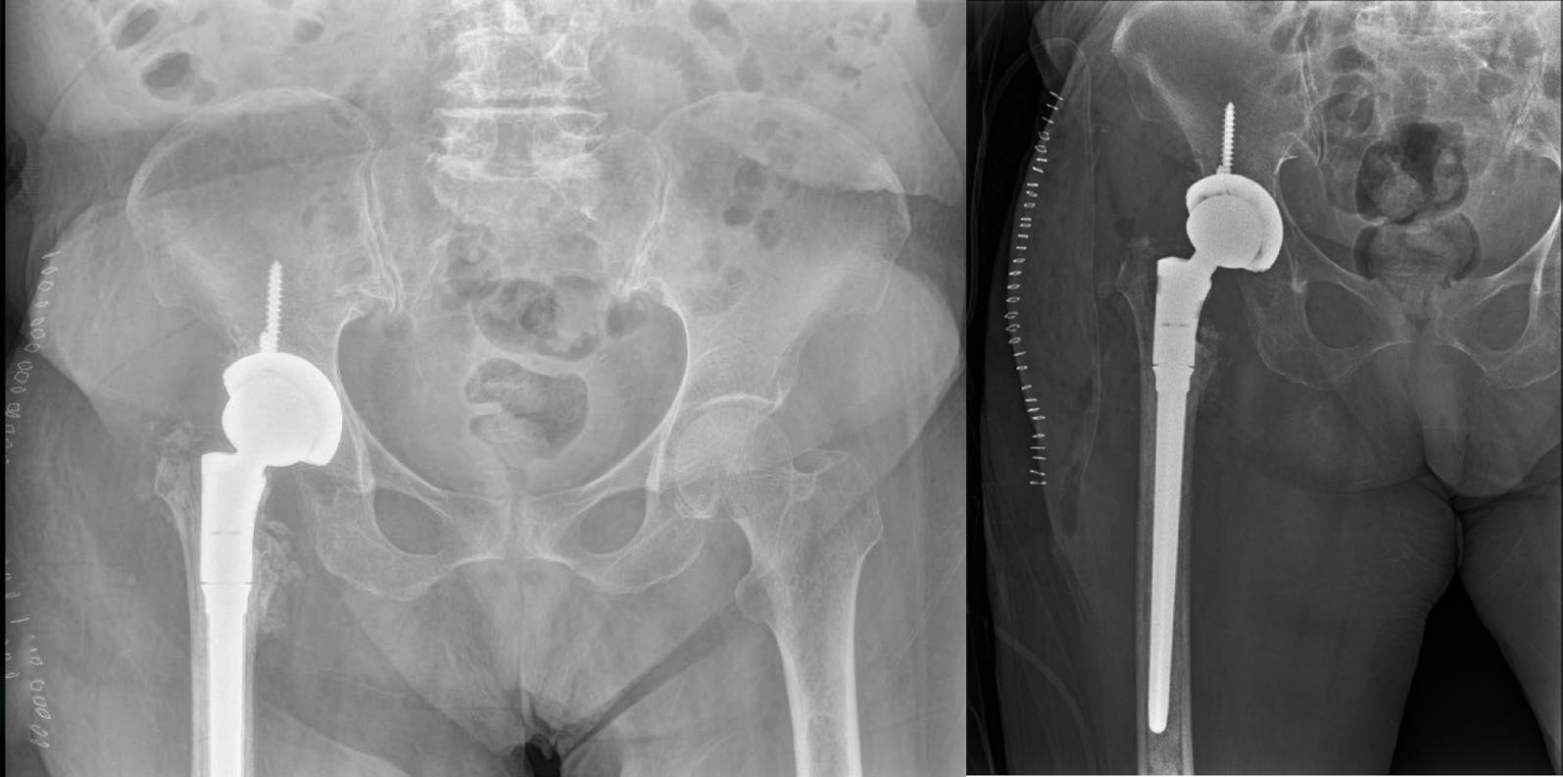
Post-revision anteroposterior pelvis radiograph demonstrating total hip arthroplasty (THA) performed after screw cut-out–related fixation failure

Postoperative infections occurred in 12 patients (3.4%) and were managed surgically with irrigation–debridement (n = 10) or implant removal with debridement (n = 2). Among patients with screw cut-out (n = 56), 51 (91.1%) underwent revision surgery. Revision procedures comprised repeat PFN fixation in 9/56 (16.1%), partial hip arthroplasty in 24/56 (42.9%), and total hip arthroplasty in 12/56 (21.4%) (Fig. 4). In addition, 5/56 (8.9%) patients underwent other revision procedures (e.g., implant removal ± debridement or limited reconstruction). The remaining 5/56 (8.9%) were managed non-operatively owing to advanced age or significant comorbidities.

## Discussion

This study demonstrates that patient-specific anatomical alignment, quantified as the absolute deviation between the postoperative and contralateral collodiaphyseal (CCD) angles (|Δ angle|), is independently associated with screw cut-out after proximal femoral nailing (PFN). Alongside the well-established parameters of tip–apex distance (TAD) and reduction quality, |Δ angle| emerged as a significant radiographic factor. These results support the concept that individualized anatomical alignment, rather than a fixed “ideal” neck–shaft angle, may provide a more accurate and clinically meaningful target for reduction.

The importance of accurate implant positioning is well recognized. Baumgaertner et al. established the TAD as the most reliable radiographic predictor of fixation failure, with values greater than 25–30 mm markedly increasing cut-out risk [8]. Subsequent large-scale studies confirmed these thresholds and validated the predictive utility of calcar-referenced TAD [7, 13]. Similarly, reduction quality remains one of the most consistent determinants of stability across multiple series [11, 23, 27, 28]. In the present cohort, both parameters were independently associated with cut-out, confirming their reproducibility across diverse surgical techniques and patient populations.

What differentiates the current study is the demonstration that restoring the CCD angle relative to the contralateral hip adds prognostic value beyond these traditional parameters. Prior research has commonly referred to a fixed target CCD angle of approximately 130°, emphasizing the avoidance of varus reduction [13, 29, 30]. Some authors have even suggested achieving a postoperative valgus alignment 5°–10° greater than the contralateral side to mitigate varus loading on the implant [31]. However, such recommendations were conceptual and not quantitatively validated in clinical outcome analyses. Rogers et al. and Walton et al. also measured contralateral CCD angles to assess anatomical variation and surgical planning reliability [32, 33], but these studies did not evaluate their relationship to mechanical failure. Likewise, Kashigar et al. compared postoperative and contralateral neck–shaft angles—termed the “cervical angle difference”—and found significance only in univariate analysis, with loss of predictive power after multivariable adjustment [13]. This likely occurred because their signed difference approach treated varus and valgus deviations as opposing values that canceled each other statistically.

From a biomechanical perspective, deviations from the native CCD alignment alter load transmission across the femoral head–neck junction. Excessive varus angulation increases compressive and shear forces on the superior femoral head, predisposing the lag screw to superior cut-out. Conversely, over-valgus reduction may shift the load medially, reducing cortical support and increasing toggle forces at the nail–screw interface. Therefore, minimizing deviation from the patient’s inherent alignment may optimize load sharing and reduce micromotion at the bone–implant interface [13, 29, 34–36].

In contrast, the present study introduced the absolute deviation (|Δ angle|) between postoperative and contralateral CCD angles, reflecting the *magnitude* of malalignment irrespective of direction. This parameter quantifies the extent of deviation from the patient’s native anatomy, allowing a truly individualized assessment of reduction accuracy. By removing directional bias, |Δ angle| provided stronger prognostic performance and remained independently associated with screw cut-out after adjustment for TAD and reduction quality. Our findings therefore validate contralateral anatomical alignment as a clinically meaningful and modifiable factor in PFN surgery. Although other indices such as the Cleveland zone or Singh Index have been explored as markers of implant position and bone quality, these were not included in the current analysis. Bone mineral density was objectively assessed through DEXA T-scores, and implant placement was quantitatively represented by TAD and reduction quality—parameters known to correlate with Cleveland zone positioning. Consequently, additional indices were deemed redundant within the current analytic framework.

Consistent with previous studies, most patients with screw cut-out required revision arthroplasty, most commonly partial hip replacement [37, 38]. These revision procedures resulted in delayed rehabilitation and inferior functional recovery, as reflected by the lower Harris Hip and Barthel scores in this subgroup. These findings emphasize that mechanical failure after PFN directly compromises patient independence and overall clinical outcomes.

Beyond the radiographic associations, this study highlights the functional implications of mechanical failure. Patients who experienced cut-out exhibited significantly lower Harris Hip Scores and Barthel Index values, as well as delayed return to full weight bearing. These findings underscore that cut-out is not merely a radiographic complication but a clinically debilitating event, compromising mobility, independence, and quality of life. Few previous studies have directly quantified this functional deterioration [39], and its confirmation here emphasizes the broader patient-centered impact of mechanical failure.

From a surgical standpoint, these results suggest that intraoperative assessment should extend beyond minimizing TAD and achieving acceptable reduction. Restoration of the CCD alignment relative to the contralateral hip should also be considered a key objective. Intraoperative fluoroscopic comparison with the uninjured side may provide a simple yet effective method to avoid excessive varus or valgus reduction. Incorporating patient-specific anatomical alignment into preoperative planning and intraoperative decision-making could therefore reduce fixation failure and improve postoperative recovery. Practically, surgeons can compare the intraoperative fluoroscopic image with a preoperative or contralateral templating radiograph to estimate the patient’s native CCD angle. Digital overlay or image mirroring techniques may assist in restoring individualized alignment, providing a feasible intraoperative safeguard against excessive varus or valgus reduction.

### Strengths and limitations

This study has certain limitations. Its retrospective and single-center design may limit generalizability. Inter- and intra-observer reliability for |Δ angle| measurement were not assessed in this study because all radiographs were evaluated by a single experienced observer. Future studies including repeated measurements by multiple observers at two timepoints are warranted to establish inter- and intra-observer agreement through ICC analysis. The moderate sample size provided adequate statistical power for multivariable analysis, but larger multicenter datasets are needed to confirm external validity and to explore potential thresholds for clinical application. Selection bias is possible given the retrospective design and exclusion of patients with incomplete imaging.

Nevertheless, this study has several notable strengths. To our knowledge, it represents the first clinical cohort to quantitatively evaluate and validate patient-specific anatomical alignment (|Δ angle|) as an independent factor associated with screw cut-out after PFN. The inclusion of validated functional outcome measures (Harris Hip Score, Barthel Index, and time to full weight bearing) provides a comprehensive perspective on both radiographic and clinical consequences of mechanical failure. Future prospective studies with larger cohorts should confirm these findings and may serve as a basis for developing predictive models that integrate individualized anatomical parameters.

## Conclusion

In conclusion, patient-specific anatomical alignment—expressed as the absolute deviation between postoperative and contralateral CCD angles (|Δ angle|)—is an independent predictor of screw cut-out after proximal femoral nailing. Along with tip–apex distance and reduction quality, |Δ angle| represents a modifiable parameter reflecting individualized anatomical restoration. Screw cut-out also correlated with poorer functional recovery, highlighting its clinical impact. Intraoperative assessment of contralateral CCD alignment may enhance reduction accuracy and reduce fixation-related complications.

## Data Availability

The datasets generated and/or analyzed during the current study are available from the corresponding author on reasonable request.

## Abbreviations

AO/OTA: Arbeitsgemeinschaft für Osteosynthesefragen / Orthopaedic Trauma Association
AP: Anteroposterior
AUC: Area under the ROC curve
BI: Barthel index
BMI: Body mass index
CCD: Collodiaphyseal (neck–shaft) angle
CI: Confidence interval
DEXA: Dual-energy X-ray absorptiometry
EPV: Events per variable
HHS: Harris hip Score
HL test: Hosmer–Lemeshow goodness-of-fit test
ICC: Intraclass correlation coefficient
IRB: Institutional review board
OR: Odds ratio
PACS: Picture archiving and communication system
PFN: Proximal femoral nailing
ROC: Receiver operating characteristic
SD: Standard deviation
TAD: Tip–apex distance
CalTAD: Calcar-referenced tip–apex distance
WB: Weight bearing
|Δ angle|: Absolute deviation between postoperative and contralateral CCD angles
